# Supplementary figures and images for: Development of a potential vaccine against Capripox virus implementing reverse vaccinology and pan-genomic immunoinformatics
Source: PLoS One. 2025 Jul 2;20(7):e0326310. doi: 10.1371/journal.pone.0326310 (PMC12221009; doi:10.1371/journal.pone.0326310)

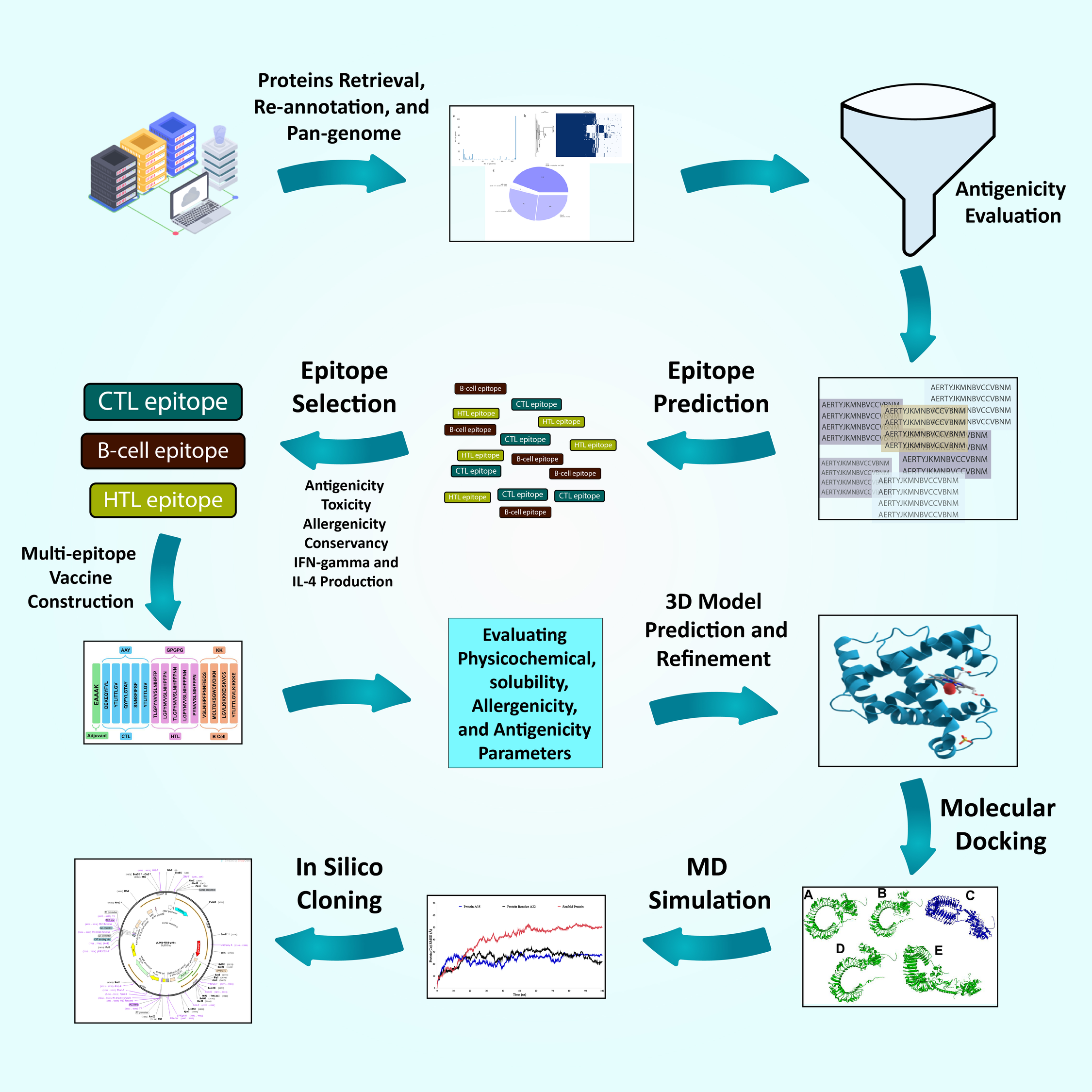

Supplement: S3 Fig — In this Fig, we exhibited the whole process of the Capripox virus vaccine design. (TIF) [file pone.0326310.s003.tif]
